# An in silico study on antidiabetic activity of bioactive compounds in *Euphorbia thymifolia* Linn.

**DOI:** 10.1186/s40064-016-2631-5

**Published:** 2016-08-18

**Authors:** T. Hoang Nguyen Vo, Ngan Tran, Dat Nguyen, Ly Le

**Affiliations:** 1 International University – Vietnam National University - HCMC, Quarter 6, Linh Trung Ward, Thu Duc District, Ho Chi Minh City, Vietnam; 2 Institute of Computational Science and Technology - HCMC, Ho Chi Minh City, Vietnam

**Keywords:** *E. thymifolia*, Diabetes mellitus, Molecular docking, Pharmacophore, LigandScout

## Abstract

Herbal medicines have become strongly preferred treatment to reduce the negative impacts of diabetes mellitus (DM) and its severe complications due to lesser side effects and low cost. Recently, strong anti-hyperglycemic effect of *Euphorbia thymifolia* Linn. (*E. thymifolia*) on mice models has reported but the action mechanism of its bioactive compounds has remained unknown. This study aimed to evaluate molecular interactions existing between various bioactive compounds in *E. thymifolia* and targeted proteins related to Type 2 DM. This process involved the molecular docking of 3D structures of those substances into 4 targeted proteins: 11-β hydroxysteroid dehydrogenase type 1, glutamine: fructose-6-phosphate amidotransferase, protein-tyrosine phosphatase 1B and mono-ADP-ribosyltransferase sirtuin-6. In the next step, LigandScout was applied to evaluate the bonds formed between 20 ligands and the binding sites of each targeted proteins. The results identified seven bioactive compounds with high binding affinity (<−8.0 kcal/mol) to all 4 targeted proteins including β-amyrine, taraxerol, 1-*O*-galloyl-β-d-glucose, corilagin, cosmosiin, quercetin-3-galactoside and quercitrin. The pharmacophore features were also explained in 2D figures which indicated hydrophobic interactions, hydrogen bond acceptors and hydrogen bond donors forming between carbonyl oxygen molecules of those ligands and active site residues of 4 targeted protein.

**Graphical abstract:**
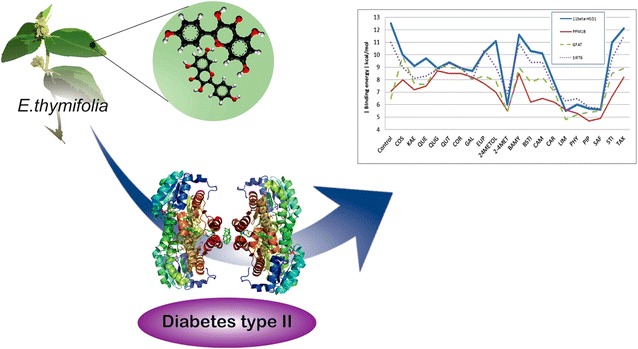
*Euphorbia thymifolia* Linn. is a small prostrate herbaceous annual weed that can positively impact on reducing hyperglycemic effect. In order to clearly understand about molecular level of the its bioactive compounds, in silico approach is performed

*Euphorbia thymifolia* Linn. is a small prostrate herbaceous annual weed that can positively impact on reducing hyperglycemic effect. In order to clearly understand about molecular level of the its bioactive compounds, in silico approach is performed

## Background

*Euphorbia thymifolia* is a small prostrate herbaceous annual weed which is abundant in waste places and open grasslands and distributes in most Asian countries. This medicinal plant has been studied for many bioactivities and therapeutic effects such as anti-microbial effect (Killedar et al. 2011), bronchial asthma (Sharma and Tripathi 1984) and the anti-hyperglycemic effect of *Euphorbiaceae* family has been fully reviewed (Bnouham et al. 2006). Besides that, *E. thymifolia* has also been traditionally used for treatment of gastrointestinal disorder, inflammatory and respiratory diseases (Loi 2015).

Diabetes mellitus (DM) and its complications are main causes of deaths in most countries. Type 2 DM has also been known as another terms “Non-insulin dependent diabetes mellitus (NIDDM)” which accounted for more than 90 % of diagnosed cases of DM in adults (International Diabetes Federation (IDF) 2015). In accordance with Ford et al. (2002), the statistics of patients suffering Type 2 DM and metabolic syndromes were estimated about 50 million in the US and 314 million around the world and this number was predicted to increase dramatically in the next decades. The feature of Type 2 DM is the partial or complete failure in using insulin (insulin resistance) even though the functional insulin is available and then causes hyperglycemia. To overcome this resistance, the pancreatic β cells produce extra mount of insulin to maintain glucose in the normal range. However, this process is only effective in the short term as burnout β cell occurs. At this time, the patients have suffered Type 2 DM.

Many efforts to figure out the effective treatments for Type 2 DM have been increased. For many years, scientists have endeavored to apply not only pharmacological methods but also non-pharmacological approaches but none of them met all safety requirements in medication. Losing weight and doing exercise have been highly recommended as two major non-drug therapies to increase insulin sensitivity. In aspect of pharmaceutical science, although metformin and thiazolidinedione both have good effect in insulin resistance, they cannot be widely used because of their undesirable side effects. Currently, research on relationships between antioxidant compounds and Type 2 DM has been well published and documented. People revealed that an intake of antioxidant in diet has contributed to reduce the development of Type 2 DM (Montonen, et al. 2004; Evans 2007). Besides, in 2005, Fraga investigated that the intake of dark chocolate which was a rich source of flavonols could decrease blood pressure and improved insulin sensitivity in healthy persons (Fraga 2005).

In the light of these evidences, the objective of this research is to test the anti-hyperglycemic activity of antioxidant compounds in the ethanolic extracts of *E. thymifolia* by using them as ligands for four targeted proteins to determine which compound is effective binder. The chemical composition analyzed by GC–MS from areal part of *E. thymifolia* suggested three main families: tannin, flavonoid and terpenoid (Sandeep et al. 2009; Prasad and Bisht 2011) which are strong anti-oxidant compounds. Possessing polyphenol structure involving high number of hydroxyl group inside, tannin and flavonoid were, thus, predicted to be able to form hydrogen bonds with various reactive oxygen species, such as singlet oxygen, peroxynitrite and hydrogen peroxide which are major causes of cell damages. Due to this mechanism, tannin and flavonoid were considered to play potential roles in reducing the oxidative stress related to Type 2 DM (Evans 2007; Maiese et al. 2007). Terpenoid is an enormous class of organic compound in plant whose potential antioxidant activity has already studied (Gonzalez-Burgos and Gomez-Serranillos 2012). However, there are no research indicating their affinity for Type 2 DM. Four targeted proteins used in this study was previously investigated to serve as potential drug target for Type 2 DM (Nguyen and Le 2012; Shi 2009; Vogel 2002). 11β-HSD1 (11β-hydroxysteroid dehydrogenase type I) or “cortisone reductase” is an NADPH-dependent enzyme highly expressed in main metabolic tissues including liver, adipose tissue, and the central nervous system. In these tissues, HSD11B1 reduces cortisone to the active hormone cortisol that activates glucocorticoid receptors. 11βHSD1 inhibition is a tempting target for the treatment of glucortinoid-associated diseases, especially of Type 2 DM (Davani, et al. 2004; Andrews and Walker 1999). Glutamine-fructose-6-phosphate amidotransferase (GFAT or GFPT) is the first and rate-limiting enzyme of the hexosamine pathway. GFAT controls the flux of glucose into the hexosamine pathway and catalyzes the formation of glucosamine 6-phosphate. The majority of glucose will enter the glycolysis pathway, with a small percentage entering the hexosamine pathway. GFPT or GFAT regulate the hexosamine pathway products. Therefore, this enzyme involved in a therapeutic target against Type 2 DM (Chou 2004). Protein-tyrosine phosphatase 1B (PTP1B) is a negative regulator of the insulin signaling pathway and is considered a promising potential therapeutic target, in particular for treatment of Type 2 DM. It has also been implicated in the development of breast cancer and has been explored as a potential therapeutic target in that avenue as well. Sirtuin-6 or Mono-ADP ribosyltransferase-sirtuin-6 (SIRT6) is a stress responsive protein deacetylase and mono-ADP ribosyltransferase enzyme encoded by the SIRT6 gene. SIRT6 functions in multiple molecular pathways related to aging, including DNA repair, telomere maintenance, glycolysis and inflammation. Promisingly, the absence of enzyme SIRT6 may lead to dramatically induced of blood sugar level (Hasan et al. 2002). The objective of this study was to display a range of bioactive compounds from all three families and determine if and how they interact with proteins that is important to Type 2 DM (Muthumani et al. 2016, Prasad and Bisht 2011, PROTA 2008) (Table 2).

## Methods

### Molecular docking

#### Receptor preparation

3D structure of 11-β HSD1, GFAT, PTP1B, SIRT6 were taken from Protein Data Bank as following 11β-HSD1 (PDB code 1XU7), GFAT (PDB code 2ZJ3), PTP1B (PDB code 4Y14) and SIRT6 (PDB code 3K35). To verify the capacity of the model in reproducing experimental observation with new ligand, all these structures were tested again at the binding site. Following this way, 11β-HSD1 (PDB code 1XU7) was tested again with molecule: NADPH dihydro-nicotinamide-adenine-dinucleotide phosphate (NDP), GFAT (PDB code 2ZJ4) was tested with 2-deoxy-2-amino glucitol-6-phosphate (AGP), SIRT6 (PDB code 3K35) with adenosine-5-diphosphoribose (APR) and PTP1B (PDB code 4Y14) with 3-bromo-4-[difluoro(phosphono)methyl]-*N*-methyl-Nalpha-(methylsulfonyl)-l-phenylalaninamide. This work was done by Autodock Vina (Trott and Olson 2009) and VMD was used for visualization (Humphrey et al. 1996).

#### Bioactive compound preparation

Most of the 3D structures of drug molecules in *E.thymifolia* were downloaded from PubChem Compound section of National Center for Biotechnology Information (NCBI) and the others were drawn by GaussView 5 (Dennington et al. 2009). Ligands during this process also being checked for Torsion count to detect currently active bonds with default settings. Importantly, amide bonds were checked and treated as non-rotatable. Ligands were also utilized to merge non-polar hydrogens. The 2D structures of 20 ligands are illustrated in Table 1.

**Table 1:**
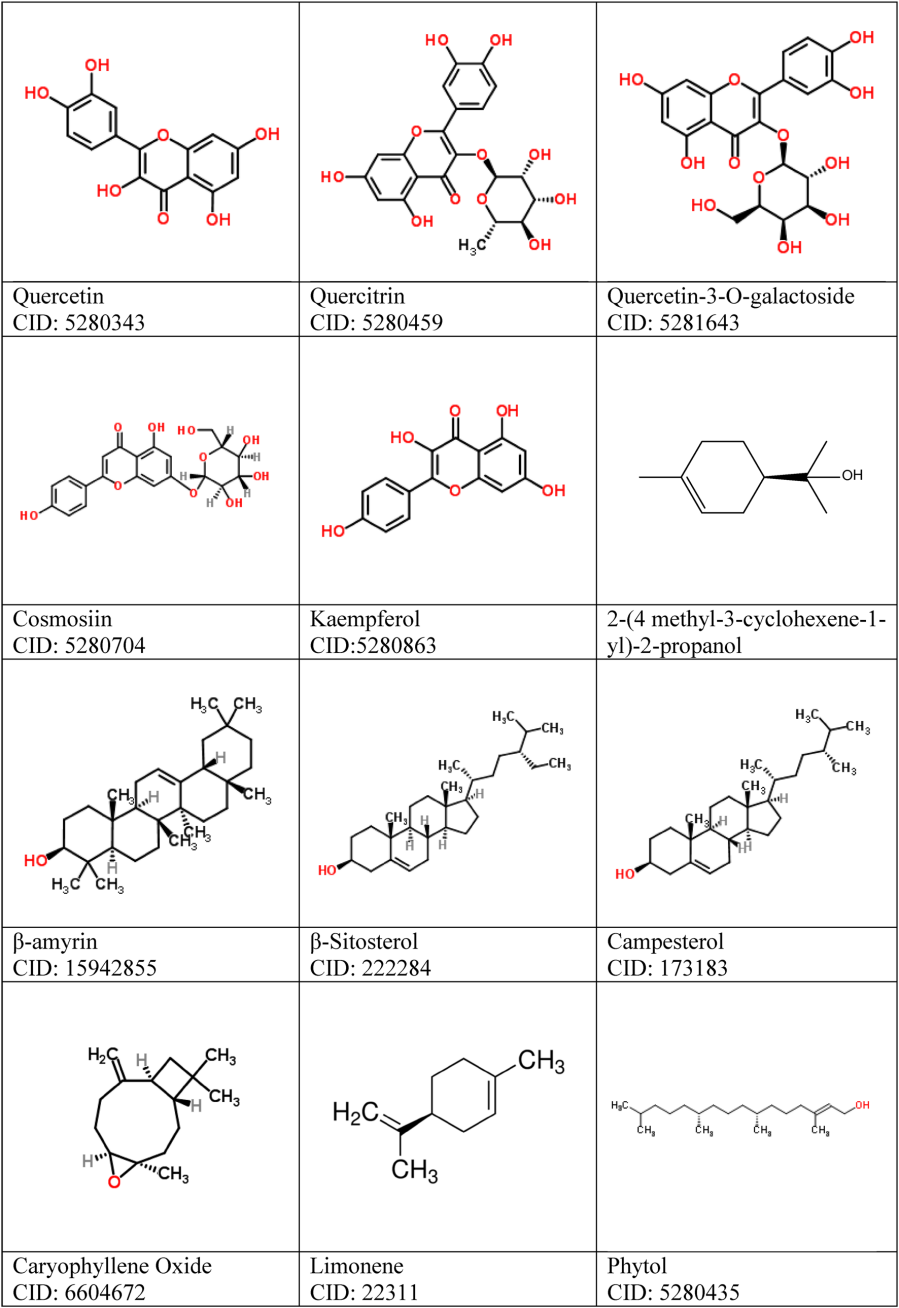

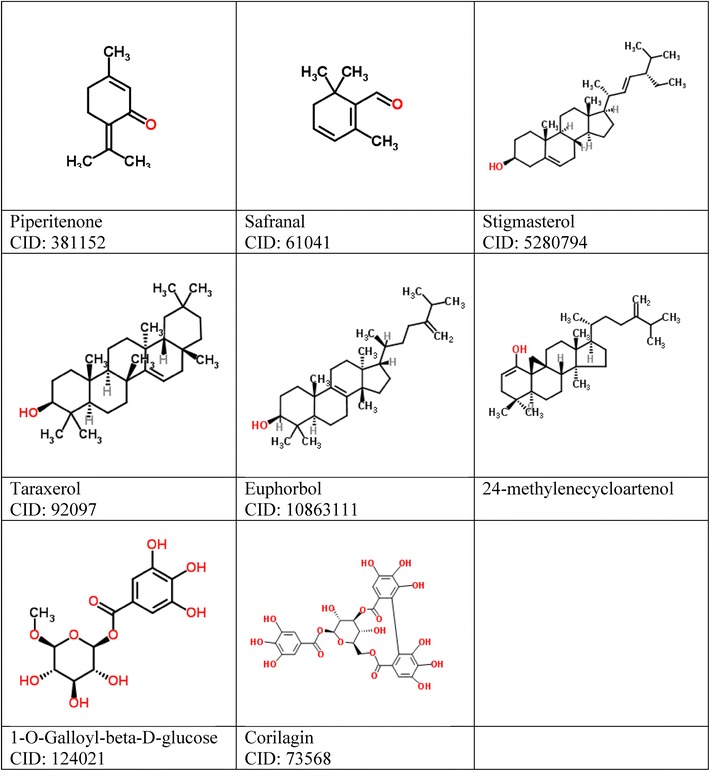
2D structures of 20 drug candidates suggested from PubChem—NCBI

#### Docking simulations

Autodock Vina (Trott and Olson 2009) was employed for binding affinity measurement. The content of configure file was determined as position of receptor file, ligand file, data of Grid-box’s three coordinates X, Y, Z were 18.125, −27.72, −0.34 respectively in case of 11β-HSD1, 8.82, 5.31, −7.903 for GFAT, −11.21, −22.77, −6.75 in PTP1B, 14.5, −18.02 and 17.04 in SIRT6, the size of Grid box which was set up in 30 × 30 × 30 points, number of modes which were 10 and the energy range which was set up at 9 kcal/mol. Docking process in AutoDock Vina has been performed with 1000 of exhaustiveness for enhancing accuracy.

### Pharmacophore analysis

This part of process was carried out by using the pharmacophore tool included in LigandScout (Wolber and Langer 2005). The program showed us the 2D and 3D structure with the position and interaction of ligand in the binding pocket of the receptor. From these 2D figures, some types of bond were identified by color and symbol. Four features namely hydrogen bond acceptor (HBA), hydrogen bond donor (HBD), negative ionizable area (NIA), hydrophobic interaction were labeled as red arrow, green arrow, red star and orange bubble (supporting information) respectively.

## Results and discussion

### Free energy binding of bioactive compound to targeted proteins

**Fig. 1:**
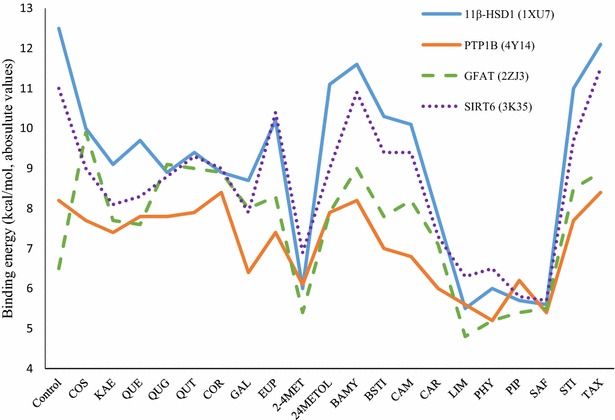
Absolute values of binding energy of 20 ligands to 4 receptors. The abbreviation of these ligands were listed as *COS* cosmosiin, *KAE* kaempferol, *QUE* Que, *QUG* quercetin-3-galactoside, *QUT* quercitrin, *COR* corilagin, *GAL* 1-*O*-galloyl-β-d-glucose1-*O*-galloyl-β-d-glucose, *EUP* euphorbol, *2-4MET* 2-(4 methyl-3-cyclohexene-1-yl)-2-propanol, *24METOL* 24 methylencycloartenol, *BAMY* Β-amyrine, *BSTI* Β-sitosterol, *CAM* campesterol, *CAR* caryophyllene oxide, *LIM* limonene, *PHY* phytol, *PIP* piperiterone, *SAF* safranal, *STI* stigmasterol, *TAX* taraxerol. Besides that, blue line represented for 11β-HSD1 protein, followed by the *purple*, *green* and *red* were labeled for PTP1B, GFAT1, SIRT6, respectively

The line chart (Fig. 1) showed the binding capacity of all three family bioactive compounds: tannin, flavonoid and terpenoid in *E. thymifolia* on 4 proteins related to Type 2 DM in humans. In this chart, tannin and flavonoid families included first seven compounds. Among those docking result, the absolute value of binding energy ranged from 7.2 to 10.4 (kcal/mol; Fig. 1). In this range, the greatest result was in five compounds of both families which were higher 8 kcal/mol in term of absolute value. Those are cosmosiin, quercetin-3-galactoside, quercitrin, corilagin, 1-O-galloyl-β-d-glucose and which were selected for pharmacophore analysis step. Besides that, kaempferol and quercetin of flavonoid family also had good results but this was different in each protein, perhaps the amino acid construction of each protein. For example, the binding affinity of quercetin was 9.7 and 8.3 kcal/mol in 11β HSD1 (1XU7) and SIRT6 (3K35) respectively, compared to 7.8 and 7.6 in PTP1B (4Y14) and GFAT1 (2ZJ3). Although there have been fluctuations in this range, the result of tannin and flavonoid were still high. This reflected the fact that the polyphenol structure with high number of hydroxyl group which serve to facilitate ligands in forming hydrogen bonds with free residue of receptor.

In addition, Fig. 1 also indicated the best receptor for these bioactive compounds in *E. thymifolia*. Following this chart, the line for 11β-HSD1 (1XU7) stayed at the upper level, followed by GFAT1 and SIRT6 at middle, and then the line of protein PTP1B (4Y14) located at bottom of chart. This proves that the 11β-HSD1 was the best receptor for binding of tannin and flavonoid family. In term of terpenoid family, 12 compounds have 3D structure on NCBI website, and their absolute value of binding energy was illustrated in Fig. 1. The good binding energy (>|−8| kcal/mol) belonged to line of 11β-HSD1 (1XU7). This line has half of result which was larger 10 kcal/mol in term of absolute value. For this reason, the 11β-HSD1 line located at top of chart. Followed by SIRT6 protein line which had 6 molecules in range of 9 and 11.5 kcal/mol, the next position is GFAT1 line and then in the bottom of chart, the PTP1B owned 10 compounds which had low results (<|−8| kcal/mol). Terpenoid family had a highest in number of ligands in this study, but there were only two compounds β-amyrine and taraxerol were chosen for pharmacophore analysis step. Half of them, 6 compounds were rejected because of low result. Those were 2-(4-methyl-3-cyclohexene-1-yl)-2-propanol, limonene, phytol, piperiterone, safranal, caryophyllene oxide. Their absolute value of binding energy to all four proteins ranged from 4.7 to 6.5 kcal/mol. They all shared a simple structure with only one ring and few hydroxyl groups outside which may explain their low binding affinity. Thus, these molecules appear to have a low capacity to form a complex with the four target proteins.

Overall, the result of this part indicated 7 compounds which had high binding capacity (|binding energy| > 8 kcal/mol) to all four receptors 11β-HSD1, PTP1B, GFAT1, SIRT6. Both tannin and terpenoid family had 2 representers, β-amyrine and taraxerol for terpenoid group, corilagin and 1-O-galloyl-β-d-glucose for tannin family. Three last compounds belong to flavonoid family, cosmosiin, quercetin-3-galactoside and quercitrin. Besides that, in three families, the line of 11β-HSD1 always stayed in highest level. It means that there is stronger interaction of ligand on this protein, compared to other three receptors. In addition, in the active site of PTP1B, GFAT1 and SIRT6, many compounds of *E. thymifolia* had stronger binding capacity than the controls and 70 % of compounds in *E. thymifolia* can interact with 11β-HSD1 by absolute value of binding energy higher 8.5 kcal/mol (Table 2). All these statistical number proved that, *E. thymifolia* is potential drug for some proteins related to Type 2 DM.

**Table 2:** Binding energy (kcal/mol) of bio-molecules in *E. thymifolia* to 11β-HSD1, PTP1B, GFAT and SIRT6

Family	Ligand	Binding energy (kcal/mol)			
		11β-HSD1 (1XU7)	PTP1B (4Y14)	GFAT (2ZJ3)	SIRT6 (3K35)
	Control	NDP: −12.5	C0A: −8.2	AGP: −6.5	APR: −11.0
Flavonoid	*Sample*				
	Cosmosiin	−10.0	−7.7	−9.9	−9.0
	Kaempferol	−9.1	−7.4	−7.7	−8.1
	Quercetin	−9.7	−7.8	−7.6	−8.3
	Quercetin-3-galactoside	−8.9	−7.8	−9.1	−8.8
	Quercitrin	−9.4	−7.9	−9.0	−9.3
Tannin	Corilagin	−8.9	−8.4	−8.9	−9.0
	1-*O*-Galloyl-beta-d-glucose	−8.7	−6.4	−8.0	−7.9
Terpenoid	Euphorbol	−10.2	−7.4	−8.3	−10.4
	2-(4 methyl-3-cyclohexene-1-yl)-2-propanol	−6.0	−6.1	−5.4	−6.9
	24 methylen cycloartenol	−11.1	−7.9	−7.9	−9.0
	β-Amyrine	−11.6	−8.2	−9.0	−10.9
	β-Sitosterol	−10.3	−7.0	−7.8	−9.4
	Campesterol	−10.1	−6.8	−8.2	−9.4
	Caryophyllene	−7.8	−6.0	−7.1	−7.3
	Limonene	−5.5	−5.6	−4.8	−6.3
	Phytol	−6.0	−5.2	−5.2	−6.5
	Piperiterone	−5.7	−6.2	−5.4	−5.8
	Safranal	−5.6	−5.4	−5.5	−5.7
	Stigmasterol	−11.0	−7.7	−8.5	−9.7
	Taraxerol	−12.1	−8.4	−8.9	−11.5

### Pharmacophore analysis

#### 11β-HSD1 and GFAT1

**Fig. 2:**
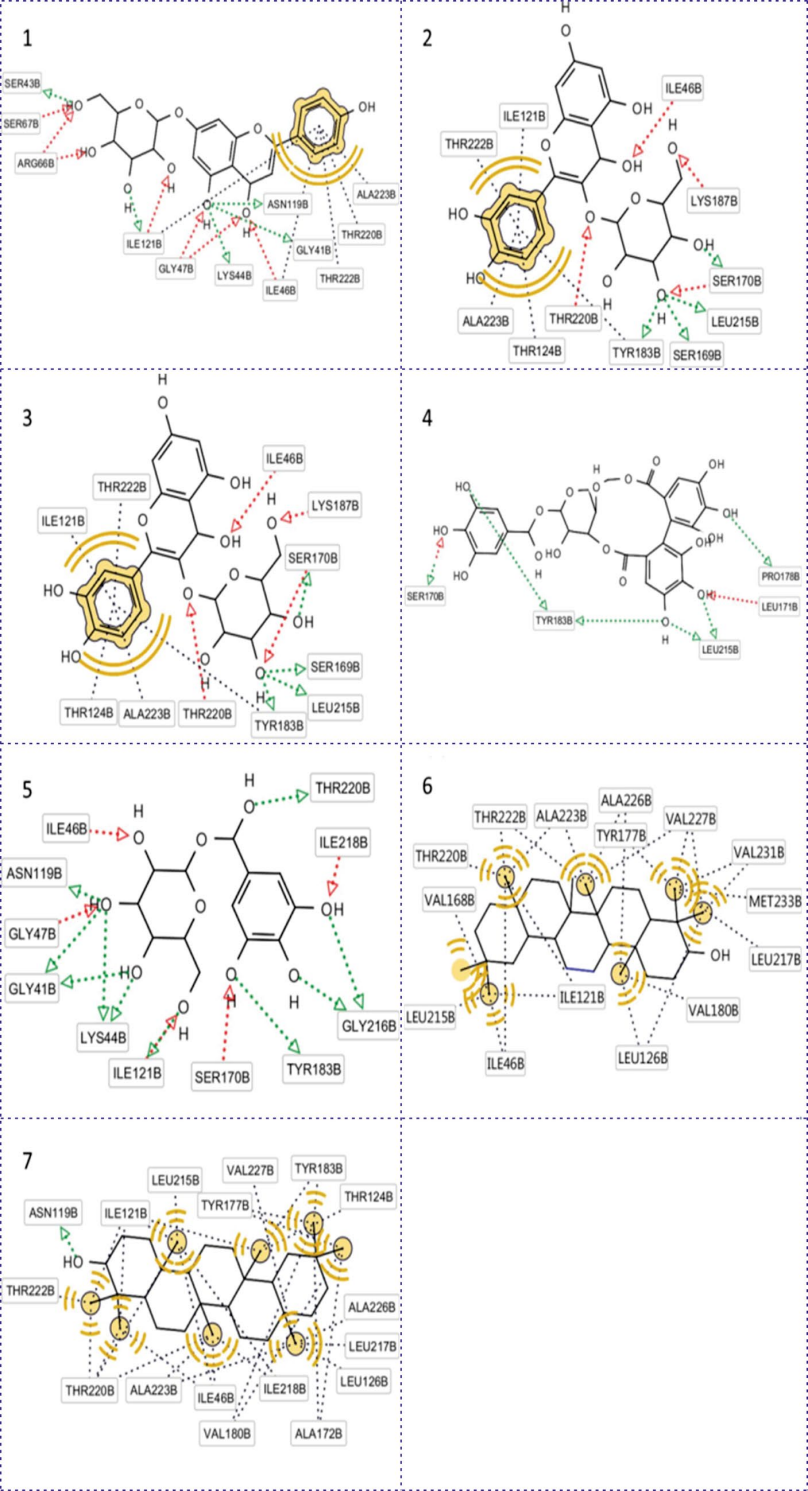
Binding modes of selective compounds with 11β-HSD1. **1** Cosmosiin, **2** quercetin-3-galactoside, **3** quercitrin, **4** corilagin, **5** 1-*O*-galloyl- β-d-glucose, **6** β-amyrine, **7** taraxerol (The *red* and *blue arrows* were hydrogen donor and receptor bonds and the *black round dot line* was hydrophobic interaction. *Yellow dot* was hydrophobic region of ligand.)

Pharmacophore analysis is an explanation step for docking result: low or high binding affinity of ligand to receptors. Five molecules of tannin and flavonoid group (1-*O*-galloyl-β-d-glucose, corilagin, cosmosiin, quercetin-3-galactoside, quercitrin) were frequently within hydrogen contact with residues Ile 46, Tyr 183, Ile 121, Ser 170 (Fig. 2). From this observation, four residues seemed to be an important substrate recognition site of 11β-HSD1. This conclusion is strongly supported by studies on crystal structures and biochemical of 11β-HSD1 (Hosfield et al. 2005; Hult et al. 2006). Especially, Ile 46 and Ile 121, both of them were dual role leading to close contact with five compounds by hydrogen bonds and also establish more hydrophobic interactions with benzene ring on ligand [Fig. 2(1, 2, 4)]. In addition, 1-*O*-galloyl-β-d-glucoseand cosmosiin could link to the receptor with a high number of hydrogen bonds compared to corilagin, quercetin-3-galactoside and quercitrin. This is proper explanation for high binding affinity of cosmosiin. This action can be explained by the affinity of each steroidal hydroxyl group for the receptor. For example, the functional group in cosmosiin could donate two or three hydrogen bonds with different residue such as Ser 43, Ser67, Arg 66, Lys 44, Gly 41, Asn 119. In tannin family, although 1-*O*-galloyl-β-d-glucose showed much stronger interaction than corilagin in term of hydrogen bond, its binding capacity was lower. To fully understand this phenomenon, molecular dynamic (MD) simulation on the complexes is suggested.

Along with hydrogen bond, hydrophobic interactions were also displayed. Β-amyrine and taraxerol seemed to be rich on hydrophobic contact at position of the methyl group which was non-polar [Fig. 2(6, 7)]. These two compounds were also in contact with this receptor because of the presence of the benzene ring. The residue Thr 124, Thr 220 and Thr 222 were three residues which could form not only hydrophobic interaction with terpenoid family but also hydrogen bond with 1-*O*-galloyl-β-d-glucose, quercetin-3-galactoside, quercitrin, members of tannin, and flavonoid group. Furthermore, in Fig. 2(2), the residues Thr 220, Thr 222, Ala 223, Ile 121, Leu 217 were frequently observed in ligand-receptor interactions between, so they could be a critical part in binding pocket. One important thing that Ser 261 and Arg 269 was shown as largely hydrophobic residues in previous study involving crystal structure analysis (Hult et al. 2006) but in the figures from our study, these hydrophobic interactions were not present.

**Fig. 3:**
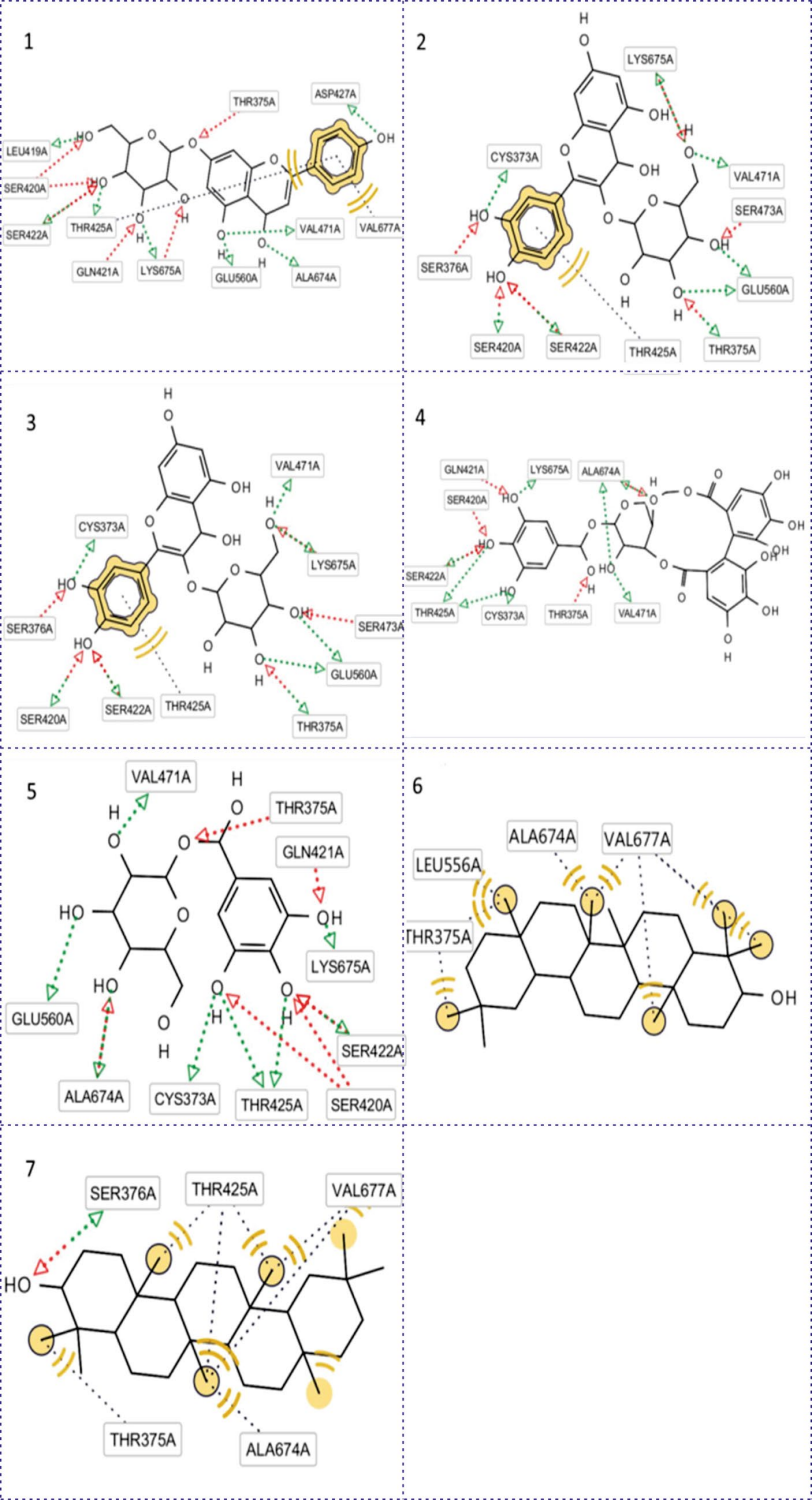
Binding modes of selective compounds with GFAT. **1** Cosmosiin, **2** quercetin-3-galactoside, **3** quercitrin, **4** corilagin, **5** 1-*O*-galloyl- β-d-glucose, **6** β-amyrine, **7** taraxerol. The *red* and *blue arrows* were hydrogen donor and receptor bonds and the *black round dot line* was hydrophobic interaction. *Yellow dot* was hydrophobic region of ligand

In term of GFAT1, this protein also had good binding energy and in some cases it had higher or equal to result of 11β-HSD1. Quercetin-3-galactoside, corilagin and cosmosiin were good illustration. Figure 3(1, 2, 3) supported this statement with high number of hydrogen bonds and hydrophobic interaction with receptor. The hydrogen bonds were established between GFAT and members of tannin and flavonoid family at position of Ser 420, Lys 675, Gln 421, Thr 375, Ser 422 in binding pocket. This was also the conclusion in case of *E.hirta* and previous article of Kuo-Chen and his partners (Chou 2004). In Fig. 3, Thr 375 and Thr 425 were especial case due to the bond they linked to receptor. This residue closed to not only methyl group but also to hydroxyl group of taraxerol and benzene ring of cosmosiin and quercetin-3-galactoside, quercitrin. Therefore, it could bind to the receptor by hydrogen and hydrophobic interaction. Besides that, hydrophobic was also displayed between Val 677, Ala 674, Thr 375 and two members of terpenoid family: β-amyrine and taraxerol.

#### SIRT6 and PTP1B

**Fig. 4:**
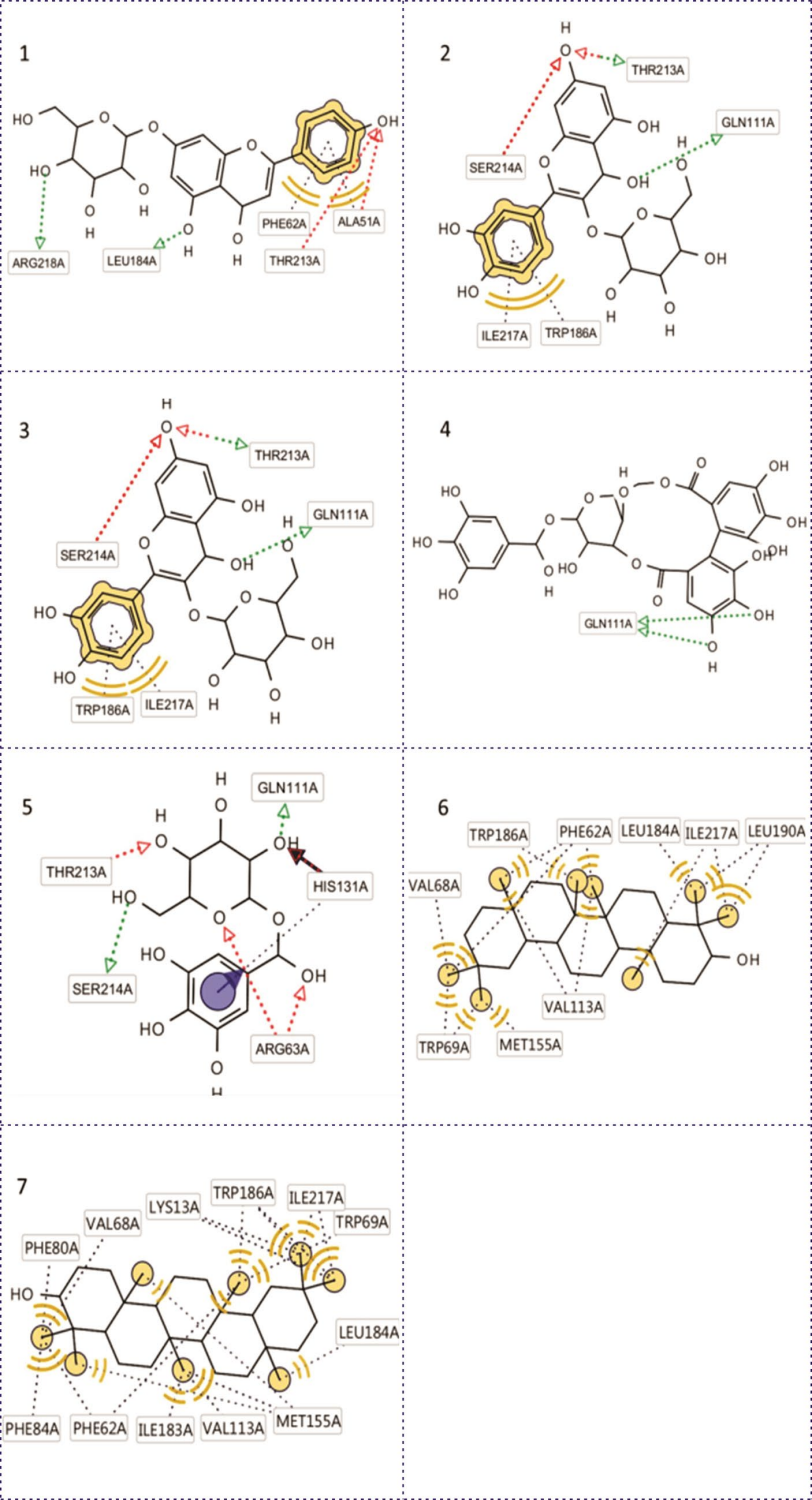
Binding modes of selective compounds with SIRT6. **1** Cosmosiin, **2** quercetin-3-galactoside, **3** quercitrin, **4** corilagin, **5** 1-*O*-galloyl-β-d-glucose, **6** β-amyrine, **7** taraxerol. The *red* and *blue arrows* were hydrogen donor and receptor bonds and the *black round dot line* was hydrophobic interaction. *Yellow dot* was hydrophobic region of ligand

1-*O*-Galloyl-β-d-glucose, corilagin, cosmosiin, quercetin-3-galactoside, quercitrin interacted with SIRT6 with the result of binding energy 8, 9, 9, 8.8, 9.3, 10.9, 11.5 in term of absolute value (Table 2). These results were smaller than 11β-HSD1. But there was a similarity with interaction of 11β-HSD1 and ligands. All these compounds can form either hydrogen bond or hydrophobic interaction with free residue in active site of SIRT6. Tannin and flavonoid family can build up hydrogen bond with Gln 111, Thr 213, Ser 214 [Fig. 4(1, 2, 3, 4, 5)]. Three residues that seem to have critical role in active site of SIRT6, but this output was totally difference in the studying of structure and biochemical function of SIRT6 of Patricia and coworker (Pan et al. 2011). This can be explained by the different tested site in our research.

In addition, the hydrophobic interactions also played an important role in docking result. The good illustration was the difference in one methyl group at carbon number 6 of rhamnoside ring (IUPAC name) of quercitrin compared to quercetin-3-galactoside structure [Fig. 4(2, 3)]. This conduct to 9.3 kcal/mol binding affinity of quercitrin compared to 8.8 kcal/mol of quercetin-3-galactoside. For this reason, this kind of bond between five of seven ligands and SIRT6 was also considerable point; these compounds form hydrophobic interaction with Ile 217, Trp186, Phe 62 at two hydrophore groups: benzene ring in flavonoid family and methyl group in terpenoid family [Fig. 4(1, 3, 6, 7)].

**Fig. 5:**
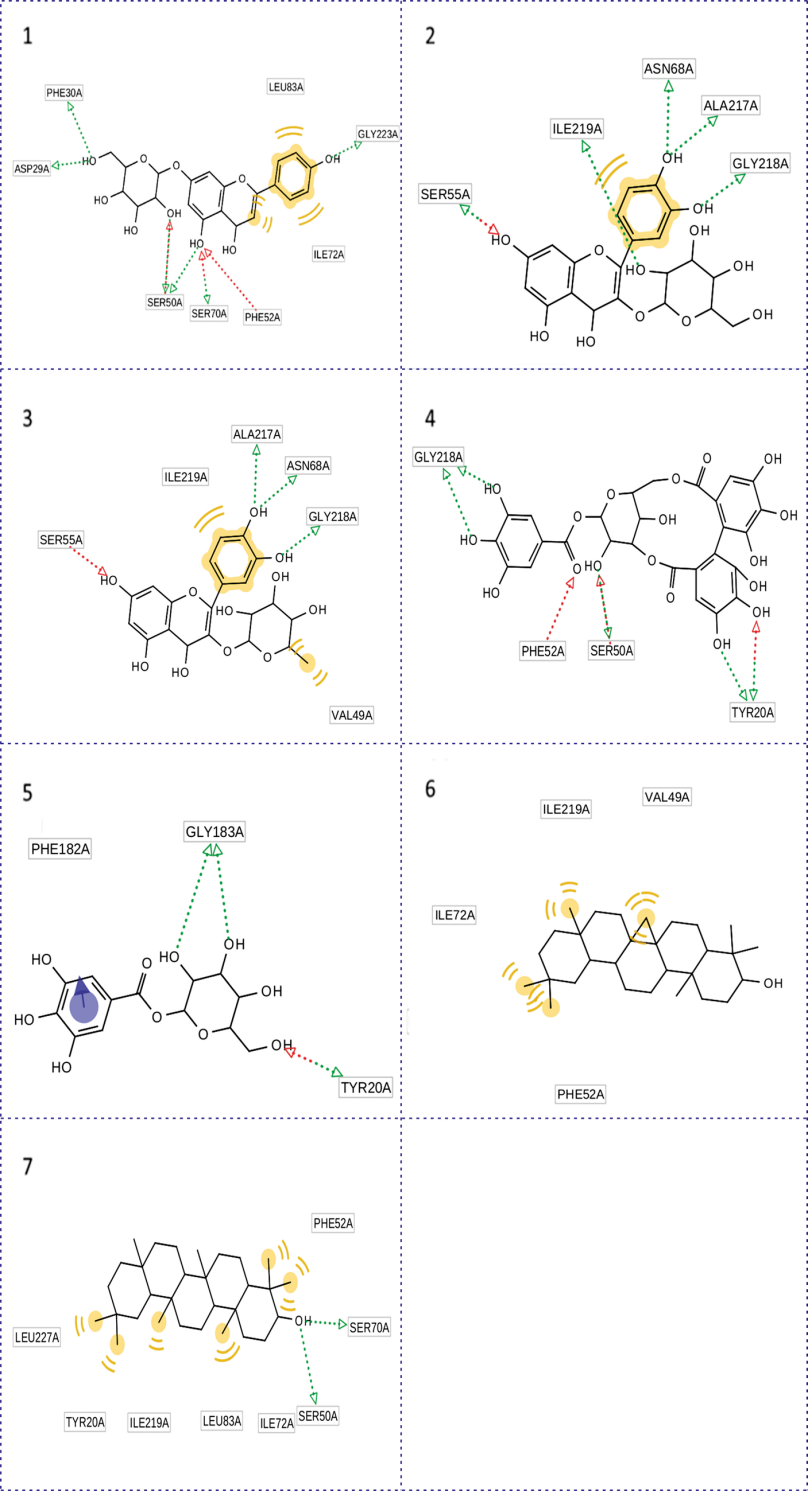
Binding modes of selective compounds with PTP1B. **1** Cosmosiin, **2** quercetin-3-galactoside, **3** quercitrin, **4** corilagin, **5** 1-*O*-galloyl-β-d-glucose, **6** β-amyrine, **7** taraxerol. The *red* and *blue arrows* were hydrogen donor and receptor bonds and the *black round dot line* was hydrophobic interaction. *Yellow dot* was hydrophobic region of ligand

The docking result of PTP1B was lower compared to three other receptors. This can be explained by the number of hydrogen bond and hydrophobic interaction in the link of ligands and SIRT6. For example, the number of hydrophobic interaction and hydrogen bond between taraxerol and four 11β-HSD1, SIRT6, GFAT1 and PTP1B were 32 [Fig. 2(7)], 23 [Fig. 3(7)], 11 [Fig. 4(7)], 8 [Fig. 5(7)] respectively, and docking results were 12.1, 11.5, 8.9, 8.4 kcal/mol respectively in term of absolute value (Table 2). In case of corilagin, the number of hydrogen bond in PTP1B was 8 [Fig. 5(4)] compared to 2 hydrogen bonds of SIRT6 [Fig. 4(4)] but the docking result was smaller. This action can be explained by the maintain time of interaction between ligand and receptors. The same with hydrogen bond, the number of hydrophobic interaction was also significantly reduced in arrangement from 11β-HSD1 to PTP1B. There were only 4 bonds between β-amyrine and PTP1B, whereas 24 bonds in case of 11β-HSD1. The duration time of the interaction between ligand and receptor is high frequency of residues Tyr 29, Phe 52, Ile 219 (Fig. 5) seem to be the significant region in active site of PTP1B.

## Conclusion

In summary, from the list of 20 compounds, seven compounds were chosen due to high absolute value of binding energy to all four receptors (>8 kcal/mol). They are β-amyrine, taraxerol, 1-*O*-galloyl-β-d-glucose, corilagin, cosmosiin, quercetin-3-galactoside and quercitrin. Polyphenol, the frame of tannin and flavonoid family had high binding affinity to all four receptors. Besides that, the binding affinity of two of the terpenoid compounds also suggested that this family is also a good prospect for the treatment of Type 2 DM.

Although the basic concepts of interaction between 20 ligands of *E. thymifolia* and 4 receptors had been already defined, many questions still remained unclear for relationship between docking result in autodock step and number of bonds in 2D structure of pharmacophore analysis step. Therefore, further research is required using, the molecular dynamic (MD) and hydrogen bond analysis to clearly determined the stability of the hydrogen bonds and hydrophobic interactions between ligands and receptors.
